# Severe Hyponatremia Due to Valproic Acid Toxicity

**DOI:** 10.14740/jocmr2219w

**Published:** 2015-07-24

**Authors:** Ena Gupta, Ryan Kunjal, James D. Cury

**Affiliations:** a Department of Internal Medicine, University of Florida College of Medicine, Jacksonville, FL, USA; b Department of Pulmonary, Critical Care and Sleep Medicine, University of Florida College of Medicine, Jacksonville, FL, USA

**Keywords:** Hyponatremia, Valproic acid overdose, Drug overdose

## Abstract

Hyponatremia is a very commonly encountered clinical entity with potentially dangerous effects and for which many precipitating factors have been identified. We present a case of valproic acid (VPA) overdose causing profound hyponatremia, with one of the lowest serum sodium levels ever documented in literature. A 54-year-old woman with hypothyroidism, hypertension and bipolar disorder presented with somnolence after intentionally ingesting 7,500 mg VPA. She was drowsy but easily arousable with no hemodynamic compromise and an unremarkable physical exam. There was no clinical suspicion for organic neurological or pulmonary disease, adrenal insufficiency or volume depletion. She was found to have a serum sodium of 99 mEq/L, low plasma osmolality (211 mOsm/kg H_2_O), and high urine osmolality (115 mOsm/kg H_2_O). Her urine sodium was 18 mEq/L. She was euthyroid (TSH: 3.06 mIU/L) and compliant with thyroxine replacement. She was admitted to the intensive care unit for close monitoring and VPA was withheld. Over 36 hours her VPA level fell from 59.3 mg/L to 22.8 mg/L, serum sodium steadily rose to 125 mEq/L and there was concomitant improvement in her mental status. At 72 hours, she was transferred for an inpatient psychiatric evaluation and her sodium level was 135 mEq/L. She luckily did not experience any seizures or decline in neurological function. The clinical presentation in this patient is consistent with the syndrome of inappropriate antidiuretic hormone secretion (SIADH) leading to a dramatic fall in sodium to a level of 99 mEq/L. Chronic VPA use has been associated with SIADH and chronic hyponatremia. Review of records in this patient from 1 year prior revealed that her last measured sodium level was 127 mEq/L. It is therefore most likely that our case is one of acute on chronic hyponatremia provoked by VPA overdose in the setting of chronic VPA use. Whilst our patient’s course was relatively benign, this case illustrates a rare consequence of VPA toxicity, which if unnoticed in another patient may be tragic.

## Introduction

Hyponatremia is defined as a serum sodium concentration below 135 mEq/L. It is classified as mild (serum sodium between 130 and 135 mEq/L), moderate (serum sodium between 121 and 129 mEq/L) and severe (serum sodium 120 mEq/L or less). The clinical presentation and optimal treatment depend not only on the absolute level of sodium concentration but also its rate of fall. As a result mild hyponatremia can cause significant symptoms if the drop in sodium level is sudden, whereas severe chronic hyponatremia can cause no symptoms due to cerebral adaption [1]. Symptoms range from no symptoms or mild vague symptoms like lethargy and nausea to severe symptoms like seizures, coma and death. Nevertheless even a mild degree of hyponatremia has been associated with an increased mortality rate as seen in a cohort study of hospitalized patients by Waikar et al in 2009 [2]. Drugs are a common cause of hyponatremia in both ambulatory and in-patient settings and are often overlooked. We present a rare case of severe hyponatremia caused by valproic acid (VPA) overdose.

## Case Report

A 54-year-old woman presented with somnolence after intentional ingestion of approximately 7,500 mg VPA. Her medical history was significant for well-controlled hypertension, hypothyroidism and bipolar disorder. Her medications included lisinopril 10 mg daily, levothyroxine 100 μg daily, paroxetine 20 mg daily, clonazepam 0.5 mg twice daily and sodium valproate 500 mg daily. At presentation she was drowsy but easily arousable and was oriented to time, place and person. She reported some nausea but no other complaints. She denied headaches or cramps and there was no witnessed seizure activity. Her physical examination was unremarkable. There were no signs of volume depletion or edema. There were no obvious central nervous system disorders, pulmonary disease, neoplasms, adrenal insufficiency, or vomiting. The patient’s initial laboratory studies in the emergency room showed serum sodium of 99 mEq/L. She was bolused 2 L of saline intravenously and admitted to the medical intensive care unit for close monitoring.

She was found to have low plasma osmolality (211 mOsm/kg H_2_O) and high urine osmolality (115 mOsm/kg H_2_O). The serum sodium valproate level at presentation was 59.3 mg/L. The urinary sodium was 18 mEq/L. Results of laboratory examination are shown in Table 1. She reported compliance with levothyroxine and was clinically euthyroid (TSH = 3.06 mIU/L). Her only previous admission was for a suicide attempt by benzodiazepine overdose. On that admission she was also noted to have hypotonic hyponatremia with serum sodium 120 - 126 mEq/L and high urine osmolality. She was advised to follow up with her primary care for the same. Of note was that she had recently been placed on VPA prior to that admission.

**Table 1:** Laboratory Examination Results on the Day of Admission

	Value	Normal range
Sodium	99	135 - 145 mmol/L
Potassium	3.7	3.3 - 4.6 mmol/L
Chloride	61	101 - 110 mmol/L
CO_2_	19	21 - 29 mmol/L
BUN	5	6 - 22 mg/dL
Creatinine	0.33	0.51 - 0.95 mg/dL
Anion gap	18	42110
Osmolality	211	290 - 308 mOsm/kg
Glucose	125	71 - 99 mg/dL
TSH	3.06	0.27 - 4.2 mIU/mL
VPA level	59.3	50 - 100 mg/L
CBC		
WBC	24	4.5 - 11 × 10^3^/mm^3^
Hemoglobin	15	12 - 16 g/dL
Hematocrit	49%	37-47%
Platelet	393	140 - 440 × 10^3^/mm^3^
Urine chemistry		
Urine osmolality	115	300 - 900 mOsm/kg
Urine sodium	18	20 mEq/L (varies with salt and water intake)

*CO_2_: carbon dioxide; BUN: blood urea nitrogen; CBC: complete blood count.

Her profound hyponatremia was attributed to VPA overdose and it was withheld. There was no further fluid administration or restriction and sodium levels were serially measured. After 36 h, the VPA level fell to 22.8 mg/L and her sodium rose steadily to 125 mEq/L. During this time she returned to her baseline mental state. She reported overdosing on VPA to help with sleep after a family dispute and had denied suicidal intent. At 72 h she was transferred out of the intensive care unit for an inpatient psychiatric evaluation and her sodium was 135 mEq/L.

## Discussion

VPA is administered as a sodium salt (13.8 mg sodium per 100 mg VPA) and therefore can produce hypernatremia in large doses. In contrast, it is a documented cause of syndrome of inappropriate antidiuretic hormone secretion (SIADH) and hyponatremia [3], but the exact mechanism by which it does so has not been fully elucidated. Nevertheless, several mechanisms have been proposed by which drugs can induce SIADH. These include stimulation of antidiuretic hormone (ADH) release by the hypophysis, enhanced ADH action on the kidney, direct action of the drug on the kidney, or inhibition of the vasopressinase activity, resulting in prolonged vasopressin half-life [4, 5]. SIADH is characterized by a euvolemic hyponatremia with concentrated urine represented by an excessive urinary sodium concentration (> 20 mmol/L) and urine hyperosmolality (> 100 mOsm/kg). These features were observed in our patient except for urine sodium of 18 mmol/L, which can vary with salt intake.

Central nervous system (CNS) dysfunction is the most common manifestation of VPA toxicity, ranging in severity from mild drowsiness to coma or fatal cerebral edema [6-8]. Indeed, hyponatremia on its own can account for similar manifestations and it remains difficult to attribute our patient’s mild symptoms to one or the other. Furthermore, it is worth noting that there has been only one other case in the literature with a reported sodium level as low as 99 mEq/L which was caused by bendroflumethiazide and sertraline-associated SIADH [9]. In spite of this profound hyponatremia, our patient was only mildly symptomatic.

We presented a case of acute on chronic hyponatremia following overdose of VPA. This patient presented with serum sodium of 99 mEq/L, decreased from the last measured serum sodium of 127 mEq/L one year prior. Long-term use of VPA was probably responsible for our patient’s chronically low sodium concentration. Subsequent overdose likely resulted in an acute drop in sodium concentration leading to this presentation. Although a dose dependent improvement of hyponatremia with reduced VPA has been noted by Branten et al in two cases [10], to date there have been no reported cases of acute severe hyponatremia due to VPA overdose as seen in our case.

It is also important to explore other factors that may have contributed to this patient’s hyponatremia. Firstly, the relationship between hypothyroidism and hyponatremia has long been described but remains highly controversial [11, 12]. Nevertheless our patient was clinically euthyroid having been compliant with L-thyroxine replacement therapy and thyroid disease was therefore an unlikely contributor to an acute drop in her sodium levels. Secondly, SIADH caused by other drugs being used by the patient, namely lisinopril and paroxetine, merit discussion. ACE inhibitors by causing increased ADH secretion have been identified as the reason for hyponatremia in a handful of cases [13]. Likewise SSRIs have been proposed to induce SAIDH with incidence rates that vary widely from 0.5% to 32% [14-16]. Moreover, our patient had been on stable doses of those medications for greater than 1 year and it is unlikely that they would have accounted for a dramatic decline in sodium level as seen on presentation. Additionally, it was the changes in VPA dosing, overdose in the first instance then being withheld in the second, which correlated most with sodium levels. While multiple factors may have contributed to low sodium levels in our patient, valproate overdose remains the most probable cause.

In summary this case describes the dramatic fall in sodium following valproate overdose to a level of 99 mEq/L, one of the lowest ever documented. Fortuitously, our patient was only mild symptomatic and had a great outcome. With increasing polypharmacy, drug-induced hyponatremia is likely to increase. Hence, while prescribing and selecting medications, their pharmacokinetic properties, their propensity for drug-drug interactions, side effect profile and toxicities should be taken into consideration and discussed with the patient. Also appropriate monitoring and follow-up should be encouraged to prevent serious health consequences.
